# Quantification of the flux of tyrosine pathway metabolites during nitisinone treatment of Alkaptonuria

**DOI:** 10.1038/s41598-019-46033-x

**Published:** 2019-07-11

**Authors:** A. M. Milan, A. T. Hughes, A. S. Davison, M. Khedr, J. Rovensky, E. E. Psarelli, T. F. Cox, N. P. Rhodes, J. A. Gallagher, L. R. Ranganath

**Affiliations:** 1 Department of Clinical Biochemistry and Metabolic Medicine, Liverpool Clinical Laboratories, Royal Liverpool University Hospital, Prescot Street, Liverpool, L7 8XP UK; 2 Department of Musculoskeletal Biology, University of Liverpool, L7 8TX Liverpool, UK; 3 National Institute of Rheumatic Diseases, Piestany, Slovakia; 4 Liverpool Cancer Trials Unit, University of Liverpool, Block C, Waterhouse Building, Liverpool, L69 3GL UK

**Keywords:** Diagnostic markers, Osteoarthritis, Bone

## Abstract

Nitisinone decreases homogentisic acid (HGA) in Alkaptonuria (AKU) by inhibiting the tyrosine metabolic pathway in humans. The effect of different daily doses of nitisinone on circulating and 24 h urinary excretion of phenylalanine (PA), tyrosine (TYR), hydroxyphenylpyruvate (HPPA), hydroxyphenyllactate (HPLA) and HGA in patients with AKU was studied over a four week period. Forty AKU patients, randomised into five groups of eight patients, received doses of 1, 2, 4 or 8 mg of nitisinone daily, or no drug (control). Metabolites were analysed by tandem mass spectrometry in 24 h urine and serum samples collected before and after nitisinone. Serum metabolites were corrected for total body water and the sum of 24 hr urine plus total body water metabolites of PA, TYR, HPPA, HPLA and HGA were determined. Body weight and urine urea were used to check on stability of diet and metabolism over the 4 weeks of study. The sum of quantities of urine metabolites (PA, TYR, HPPA, HPLA and HGA) were similar pre- and post-nitisinone. The sum of total body water metabolites were significantly higher post-nitisinone (p < 0.0001) at all doses. Similarly, combined 24 hr urine:total body water ratios for all analytes were significantly higher post-nitisinone, compared with pre-nitisinone baseline for all doses (p = 0.0002 – p < 0.0001). Significantly higher concentrations of metabolites from the tyrosine metabolic pathway were observed in a dose dependant manner following treatment with nitisinone and we speculate that, for the first time, experimental evidence of the metabolite pool that would otherwise be directed towards pigment formation, has been unmasked.

## Introduction

Alkaptonuria (AKU) (OMIM #203500) is caused by a genetic deficiency of homogentisate dioxygenase (HGD) (EC 1.13.11.5), and is characterised by high circulating homogentisic acid (HGA)^1,2^. Excretion of HGA in the urine helps maintain lower circulating HGA but inevitably HGA is also converted to a pigment in body tissues through a process termed ochronosis^1,2^. The excretion of HGA in urine tends to be protective, decreasing the bodily retention of HGA, except that its excretion can lead to kidney stones and renal failure^3,4^.

Despite the efficient renal excretion of HGA, circulating HGA also remains measurable^3,4^. Circulating HGA can be oxidised via benzoquinone acetic acid to a brown-black pigment^5^. This pigment appears ochre-coloured under haematoxylin and eosin staining, leading to the term ochronotic pigment. This ochronotic pigment alters connective tissue properties leading to tissue breakdown^6^. Most of the devastating consequences of AKU in the form of premature arthritis, cardiac valve disease, osteoporosis, fractures, muscle, ligament and tendon ruptures are secondary to ochronosis^7–9^. Despite the crucial importance of ochronosis, little is known about the circulating concentration of HGA which results in pigmentation.

Nitisinone inhibits p-hydroxyphenylpyruvate dioxygenase (EC 1.13.11.27), the enzyme leading to the formation of HGA^10,11^ and has been used for more than twenty years in the treatment of hereditary tyrosinaemia type-1 (HT-1) (OMOM #276700)^10,11^. In AKU mice, nitisinone has been shown to decrease circulating HGA^12,13^ and to inhibit ochronosis^12,13^. The decrease in HGA caused by nitisinone, both in serum and urine, is dose-dependent^14^.

HGA and tyrosine concentrations have been quantified in AKU before and after treatment with nitisinone in the dose-response study SONIA 1 (Suitability of Nitisinone in Alkaptonuria)^14^. It is clear that HGA, after being formed, can either be excreted in the urine, remain elevated in total body water, be tissue bound or be converted to ochronotic pigment. By blocking or reducing the formation of HGA, nitisinone should therefore decrease urinary HGA, total body water HGA as well as HGA-pigment polymer.

The aim of this study was to determine if the amounts of excreted metabolites (urinary) and total body water tyrosine metabolites (circulating) changed on nitisinone therapy, thereby reducing the load potentially directed towards pigment formation. We have tested this hypothesis in AKU patients with different doses of nitisinone as part of the SONIA 1 trial^14^.

## Subjects and Methods

The study was a randomised, open-label, parallel-group design with a no-treatment control group. Patients were randomised to receive either 1 mg, 2 mg, 4 mg or 8 mg nitisinone once daily or no treatment (control). Forty patients were randomised, equally distributed among the groups (eight patients per group; stratified by each study centre using randomly permutated blocks). The treatment period was of 4 weeks duration, during which the study drug, nitisinone, was administered. No details of dose, TYR or HGA results were available to medical monitors, sponsor personnel or study site personnel until study completion. Further details of the SONIA 1 study have been published in full elsewhere^14^. Patients were requested to maintain stable dietary habits during the 4-week study period in order not to change their dietary protein intake and to maintain a stable weight. Body weights were measured at each visit to provide an indication of change in diet or health (catabolism). Urine urea nitrogen was also calculated from urea measurements to further confirm that diet and health were stable over the 4 weeks of study. Urine creatinine measurements in patients across visits were checked to ensure that any differences in metabolites were not due to differences in efficiency of urine collection. Twenty four hour serum profiles of TYR were also measured to determine whether there was significant diurnal variation.

The choice of daily doses used in the present study, namely 0, 1, 2, 4 and 8 mg, was based on previous experience and gaps that existed regarding the HGA-lowering effect of nitisinone in AKU^15^. The investigational medicinal product, a 4 mg/mL suspension of nitisinone (Orfadin, Sweden) was administered in the morning and allowed easy administration of the selected doses. The daily dosing frequency in our study was based on the long half-life of nitisinone.

Written consent was obtained from all patients before any study procedures. An external data and safety monitoring board was assigned to evaluate the safety data.

### Chemical analysis

#### Urine sample collection and handling

At baseline, and week 4, urine was collected over 24 h into 2.5 L bottles containing 30 mL of 5 N sulphuric acid and stored away from bright light in cool conditions. The weight of the collected urine was recorded and used as the volume in the calculations of 24 hr urine excretion assuming a density of 1 g/mL. Aliquots of the urine were frozen and retained at −80 °C until analysis. Measurements of 24-hour urine PA, TYR, HPPA, HPLA and HGA concentrations (designated uPA_24_, uTYR_24_, uHPPA_24_, uHPLA_24_ and uHGA_24_) were performed at baseline (week 0) and week 4.

#### Serum sample collection and handling

Measurement of serum PA, TYR, HPPA, HPLA and HGA concentrations (designated sPA, sTYR, sHPPA, sHPLA and sHGA) were performed at baseline (week 0) and week 4. At each visit, one sample was collected pre-dose in fasting patients. Blood samples were collected in plain serum tubes (Sarstedt, Germany). An aliquot of serum was immediately acidified using perchloric acid (10% v/v 5.8 M), based on previous experience^16,17^ of stabilising HGA, and kept frozen at −80 °C until analysis.

Serum samples were collected across a 24 hour period at 0, 0.5, 1, 2, 3, 4, 6, 8, 10, 12, 15, 18 and 24 hours (8am was time zero as a fasting baseline) at week 0 and at week 4, from all 40 patients. Serum was processed as described above.

Samples from Piestany were transported frozen by courier to Liverpool and all biochemical analyses were performed in the Department of Clinical Biochemistry, Liverpool Clinical Laboratories, Royal Liverpool and Broadgreen University Hospital NHS Trust.

#### Analyses of PA, TYR, HPPA, HPLA and HGA

The concentrations of PA, TYR, HPPA, HPLA and HGA in serum and urine were measured by liquid chromatography tandem mass spectrometry^16,18^. The published method was further validated to include PA, HPPA and HPLA (unpublished data). All analyses were performed on an Agilent 6490 Triple Quadrupole mass spectrometer with Jet-Stream electrospray ionisation coupled with an Agilent 1290 Infinity II Ultra High Performance Liquid Chromatography pump and autosampler. Briefly this method incorporates reverse-phase chromatographic separation on an Atlantis dC18 column (100 mm × 3.0mm, 3 µm, Waters); initial chromatographic conditions of 80:20 water:methanol with 0.1% formic acid (v/v) increased linearly to 10:90 over 5 min. Matrix-matched calibration standards and quality controls were used with appropriate isotopically-labelled internal standards with quantitation in multiple reaction mode (PA and TYR in positive ionisation and HPPA, HPLA and HGA in negative ionisation). Sample preparation was by dilution in a combined internal standard solution containing ^13^C_6_-HGA, d_4_-TYR and d_5_-PA in 0.1% formic acid (v/v) in deionised water. Concentrations were adjusted to reflect the concentration differences between serum and urine samples. No internal standard was available for HPPA and HPLA at time of analysis and so ^13^C_6_-HGA was validated for use as the internal standard.

### Analysis of urine creatinine and urea

Urea and creatinine in urine were measured using colorimetric assays on a Roche Cobas c701 photometric assay system (Roche, UK).

### Total body water (W) metabolites of phenylalanine, tyrosine, hydroxyphenylpyruvate, hydroxyphenyllactate and homogentisate (_W_PA, _W_TYR, _W_HPPA, _W_HPLA, _W_HGA)

Since PA and TYR and their metabolites are small molecules that are distributed in total body water^19–21^, the concentration of circulating metabolites were corrected using the factor of 0.6 times body weight in kilograms^19–21^. Serum measurements were taken at time points throughout the day; to enable a robust mean serum concentration for each analyte, area under the curve was calculated using the trapezoidal rule.

### Total urinary metabolites

TYR and PA and their metabolites were quantitated and adjusted for the 24-hour urine volumes to yield daily metabolite excretion, before and after treatment with nitisinone.

### Statistical analysis

Continuous variables are presented using mean and standard deviation (SD). Changes in mean between pre and post-nitisinone treatment for all dose groups were assessed using a paired samples t-test. Analyses were performed using Graphpad InStat 3 software (version number 3.1); *p* values < 0.05 were considered statistically significant.

### Method approval

All methodologies used within this study were approved by members of the DevelopAKUre consortium and all data are monitored by the contract research organisation PSR Group (Amsterdam) as required by the study protocol. Patient recruitment was at two sites and as such ethical approval was provided by both The Royal Liverpool and Broadgreen University Hospital NHS Trust and National Institute of Rheumatic Diseases, Slovakia.

## Results

### Patients and study treatment

Forty patients with AKU from the clinical study sites in Piestany (n = 25) and Liverpool (n = 15), respectively, were randomised into five groups (0, 1, 2, 4 or 8 mg daily doses). The patient demographics and baseline characteristics were similar across the five groups (Table 1). 67.5% were male. The mean age of all groups was 47.2 (SD: 11.9) years. Thirty-seven were Caucasian and three were Asian. All patients had normal renal function (eGFR greater than 60 mL/min).

There was no change in body weight in the various dose groups over the 4 weeks of study (pre and post dose), consistent with having no major change in diet amongst the patients (Table 2). In addition there was no change in urine creatinine concentrations demonstrating consistent 24-hour urine collection and no change in urine urea showing no apparent change in diet in the various groups over the 4 weeks of study (pre-and post nitisinone) (Table 1).

**Table 1:** 24 hour urine metabolites in SONIA 1 in pre and post nitisinone at doses of 0, 1, 2, 4 and 8 mg daily.

		Nitisinone dose in mg daily				
		0	1	2	4	8
Age (years)		45.9 (15.2)	44.4 (10.9)	43.9 (13.7)	47.3 (10.7)	54.4 (7.3)
Female n (%)		4 (50)	1 (12.5)	3 (37.5)	3 (37.5)	2 (25)
Creatinine (mmol/24 hr)	Pre	12.1 (3.8)	17.2 (5.2)	10.9 (2.5)	13 (3.8)	11.1 (2.8)
	Post	12.3 (4.9)	16.6 (2.2)	12.9 (4.0)	14.8 (4.9)	11.4 (2.2)
Urea (mmol/24 hr)	Pre	338 (74)	441 (139)	348 (100)	413 (132)	308 (63)
	Post	360 (101)	384 (75)	343 (87)	389 (148)	328 (91)
Phenylalanine (µmol/24 hr)	Pre	97.8 (45.1)	103 (52)	86.5 (40.4)	83.2 (21.0)	101 (96.7)
	Post	96.2 (51.4)	65.0 (24)*	58.9 (21)*	71.2 (19.2)	78 (90.5)**
Tyrosine (µmol/24 hr)	Pre	112 (55)	125 (51)	103 (35)	86 (25)	129 (66)
	Post	127 (103)	1204 (334)****	1310 (636)***	1415 (576)****	1727 (2041)*
Hydroxyphenylpyruvate (µmol/24 hr)	Pre	<50	<50	<50	<50	<50
	Post	<50	18422 (6028)****	16827 (6329)****	28122 (16849)***	16623 (8890)**
Hydroxyphenyllactate (µmol/24 hr)	Pre	<20	<20	<20	<20	<20
	Post	<20	14127 (3581)****	13198 (3240)****	21455 (13256)***	13853 (2432)****
Homogentisate (µmol/24 hr)	Pre	31644 (5533)	39263 (14624)	33476 (7775)	37563 (14278)	28907 (5383)
	Post	32970 (4978)	4175 (1831)***	1706 (877)****	775 (457)****	155 (55)****
Sum metabolites (µmol/24 hr)	Pre	31854 (5584)	39490 (14775)	33666 (7859)	37733 (14307)	29137 (5541)
	Post	33193 (5063)	37994 (8529)	33099 (9625)	51838 (29541)	32436 (12987)
Sum/Creat ratio metabolites	Pre	2846 (883)	2286 (314)	3094 (323)	2838 (364)	2721 (615)
	Post	3006 (919)	2266 (287)	2630 (689)	4054 (3764)	2808 (615)

(Values expressed as Mean (SD); * < 0.05, ** < 0.01, *** < 0.001, **** < 0.0001).

**Table 2:** Serum and total serum metabolites in SONIA 1 in pre- and post nitisinone at doses of 0, 1, 2, 4 and 8 mg daily. (* < 0.05, ** < 0.01, *** < 0.001, **** < 0.0001).

		Nitisinone dose in mg daily				
Serum & Total Body Water		0	1	2	4	8
Total Body Weight (Kg)	Pre	70 (23)	88 (16)	75 (11)	77 (14)	81(14)
	Post	70 (23)	88 (17)	76 (12)	77 (15)	82(13)
**Serum measurements**						
Phenylalanine (µmol)	Pre	60 (5)	56 (5)	59 (6)	63 (9)	60 (3)
	Post	62 (6)	57 (8)	57 (8)	58 (14)*	63 (9)
Tyrosine (µmol)	Pre	60 (10)	56 (4)	64 (12)	60 (10)	60 (10)
	Post	60 (10)	672 (109)****	731 (120)****	800 (126)****	856 (106)****
Hydroxyphenylpyruvate (µmol)	Pre	<10	<10	<10	<10	<10
	Post	<10	49 (8)***	52 (19)****	57 (8)****	65 (14)****
Hydroxyphenyllactate (µmol)	Pre	<5	<5	<5	<5	<5
	Post	<5	41 (10)**	48 (9)****	64 (18)**	84 (19)****
Homogentisate (µmol)	Pre	37 (14)	34 (7)	37 (11)	38 (8)	37 (8)
	Post	36 (12)	4 (2)**	3 (2)****	ND****	ND****
**Sum serum metabolites (µmol)**	Pre	156 (23)	146 (9)	159 (20)	162 (20)	158 (16)
	Post	155 (18)	821 (115)****	891 (128)****	983 (149)****	1065 (124)****

ND refers to not determined as below lower detection limit of assay.

**Figure 1:**
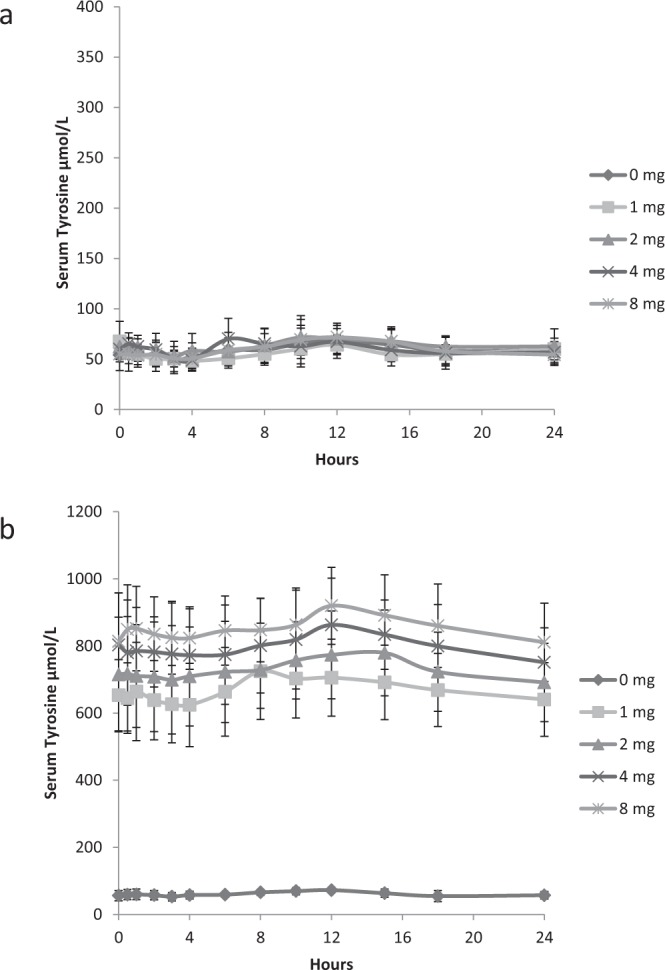
24-hour profiles of serum TYR [Mean(SD)] concentrations in SONIA 1 at baseline (**a**) and following treatment for 4 weeks with 0, 1, 2, 4 and 8 mg of nitisinone. (**b**) (Note y axis are different scales).

The 24 hour serum profile of sTYR at nitisinone doses of 0, 1, 2, 4 and 8 mg daily for 4 weeks is shown in Fig. 1. At baseline there is no significant difference between the groups, with a peak between 10–15 hours post sampling (equivalent to 18:00 to 23:00) reflecting protein load from an evening meal. At week 4 the previously described rise in sTYR, post nitisinone at all doses was evident^14^. The week 4 profile demonstrates the similar trend as seen pre-nitisinone with a peak at 10–15 hours post sampling. There was no significant dose dependent difference seen at week 4.

### 24 hour urine measurements

uPA_24_ decreased post-nitisinone significantly at 1, 2 and 8 mg doses over the 4 weeks of investigation (Table 1). uTYR_24_ was significantly higher post-nitisinone at 1, 2, 4 and 8 mg (p < 0.05 at all doses) compared with pre-nitisinone values as expected with concurrent rise in sTYR. Similarly uHPPA_24_ and uHPLA_24_ increased markedly post-nitisinone at 1, 2, 4 and 8 mg (<0.01 at all doses) compared with baseline values, where it is undetectable. uHGA_24_ decreased markedly in all patients for all doses of nitisinone, compared with pre-nitisinone values, as has previously been published^14^.

The data presented within this paper details an exploratory objective of the clinical trial with all primary objectives previously reported^14^.

### Serum (s) and total body water (w) PA/TYR metabolites

**Figure 2:**
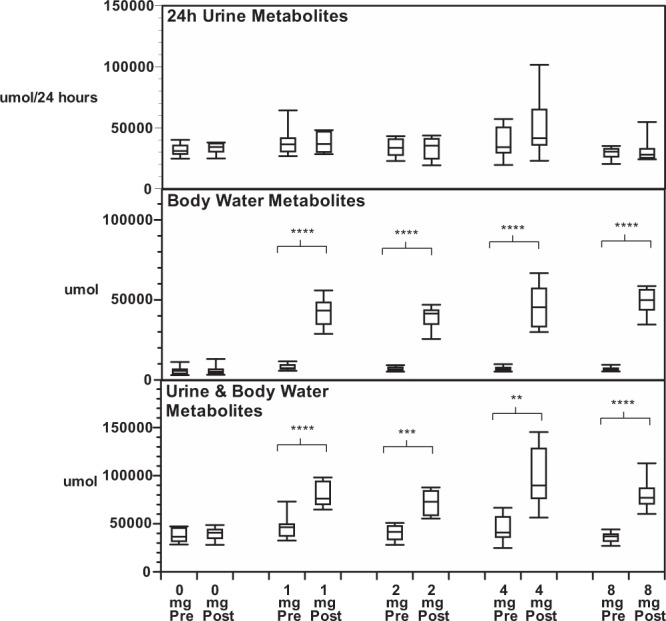
Metabolites pre and post nitisinone. Panel A shows sum of metabolites in 24 hour urine pre- and post nitisinone at doses of 0, 1, 2, 4 and 8 mg daily (µmol/24 hr). Panel B shows sum of total body water metabolites pre- and post nitisinone at doses of 0, 1, 2, 4 and 8 mg daily (µmol). Panel C shows sum of metabolites in 24-hour urine plus total body water metabolites pre- and post nitisinone at doses of 0, 1, 2, 4 and 8 mg daily. Values expressed as boxplots (25–75%) with interquartile range (5 and 95%).

Total body water metabolites were calculated using a formula that assumes water accounts for 60% of total body weight^19–21^. There was no significant difference in total body water metabolites in those patients that did not receive nitisinone (Table 3, Fig. 2). In those that received nitisinone there was no change in serum and total body water PA post-nitisinone (all p values > 0.05). However, sTYR (_W_TYR), sHPPA (_W_HPPA) and sHPLA (_W_HPLA) increased post-nitisinone using 1, 2, 4 and 8 mg doses compared with pre-nitisinone (all p < 0.01). Furthermore, sHGA (_W_HGA) decreased post-nitisinone compared with baseline at all doses (all p < 0.01).

**Table 3:** Summated metabolites and ratios in SONIA 1 in 24 hour urine, total body water and 24-hour urine plus total body water, pre and post nitisinone at doses of 0, 1, 2, 4 and 8 mg daily. (* < 0.05, ** < 0.01, *** < 0.001, **** < 0.0001).

		Nitisinone dose in mg daily				
Summated calculations		0	1	2	4	8
Sum 24 hour urine metabolites µmol	Pre	31854 (5584)	39490 (14775)	33666 (7859)	37732 (14307)	29137 (5540)
	Post	33193 (5063)	37994 (8528)	33099 (9624)	51838 (29541)	32432 (12987)
Sum total body water metabolites µmol	Pre	6555 (2622)	7741 (1607)	7135 (1425)	7485 (1773)	7678 (1425)
	Post	6623 (2768)	43484 (10358)****	40232 (7019)****	45717 (11935)****	52084 (10043)****
Difference in Sum total body water metabolites µmol		N/A	35743 (9167)	33097 (6581)	38231 (10366)	44406 (8956)
Sum ALL metabolites µmol	Pre	38409 (7102)	47232 (15745)	40801 (8916)	45218 (15935)	36815 (6155)
	Post	39815 (6971)	81476 (14052)****	73331 (14475)****	97555 (32987)***	84520 (21485)****
Difference in Sum ALL metabolites (Post – Pre) µmol		1406 (5474)	34246 (12608)	32530 (13910)	52337 (27165)	47705 (18570)
Ratio of Pre/Total in Sum ALL metabolites		0.97 (0.14)	0.58 (0.13)	0.57 (0.15)	0.48 (0.12)	0.45 (0.09)
New metabolites post-nitisinone ratio		0.05 (0.17)	0.81 (0.37)	0.85 (0.40)	1.26 (0.78)	1.32 (0.51)
New metabolites post-nitisinone %		4.8 (17)	80.8 (36.7)	84.6 (39.8)	126 (78)	132 (51)

### Summation of excreted and circulating metabolites

The sum of all urine metabolites proximal to HGA in the pathway (uPA_24_, uTYR_24_, uHPPA_24_, uHPLA_24_ and uHGA_24_) were similar pre- and post-nitisinone (Table 3, Fig. 2). Likewise, the sum of uPA_24_/creatinine, uTYR_24_/creatinine, uHPPA_24_/creatinine, uHPLA_24_/creatinine, and uHGA_24_/creatinine, were no different pre- and post-nitisinone at all doses (p > 0.05 at all doses).

The sum of _W_PA, _W_TYR, _W_HPPA, _W_HPLA and _W_HGA was significantly higher post-nitisinone, at 1, 2, 4 and 8 mg doses (all p < 0.0001).

Similarly, the combined metabolites (_ALL_PA, _ALL_TYR, _ALL_HPPA, _ALL_HPLA, and _ALL_HGA), were significantly higher post-nitisinone, compared with the pre-nitisinone baseline at 1, 2, 4 and 8 mg (all p < 0.01).

The quantity of additional total body water and ALL metabolites present at doses of 1, 2, 4 and 8 mg were also compared both as a ratio of the quantity of pre-nitisinone metabolites and as a percentage (data shown in Table 3). The ratio of pre-nitisinone ALL metabolites to post-nitisinone ALL metabolites, was 0.97, 0.58, 0.57, 0.48 and 0.45 at 0, 1, 2, 4 and 8 mg daily, respectively. The difference between post-nitisinone and pre-nitisinone ALL values when expressed as a percentage of the pre-nitisinone was +5, 81, 85, 126 and 132% at 0, 1, 2, 4 and 8 mg, respectively.

## Discussion

uHGA_24_, sTYR and sHGA results have been discussed in a previous publication on the SONIA 1 study^14^. The novel aspects of this manuscript relates to the quantification of metabolites of PA and the TYR pathway up to the point of the metabolic block observed in AKU. There has been to our knowledge no previous study examining the PA/TYR pathway metabolite flux, including all intermediates such as HPPA and HPLA, in AKU, both in disease and post-treatment with nitisinone.

### Consistency of results

It is important to note that there was no change in body weight, urine creatinine or urine urea in the various dose groups over the 4 weeks of study (pre- and post-dose), consistent with there being no determinable change in diet amongst patients over 4 weeks and also showing consistent 24 hour urine collections in SONIA 1. These factors therefore could not account for the significant differences quantitated pre- and post-nitisinone.

### Lack of overall change in urinary metabolites

It is noteworthy that the ALL metabolites in 24 hour urine were similar pre- and post-nitisinone at all doses. This suggests that while the 24 hour urine excretion of HGA was decreased by nitisinone, this was fully counter-balanced by an increase in 24 hour urine TYR, HPPA and HLPA, leading to no net difference in the quantity of these metabolites pre- and post-nitisinone. This is in keeping with the relatively free renal excretion of metabolites HPPA, HPLA and HGA and the maintained renal function during the four week trial.

### Significance of derived total body water metabolites

There was a significant increase in serum TYR, HPPA and HPLA not balanced by the decrease in serum HGA post-nitisinone. Being small molecules their distribution was adjusted for whole body water employing the calculation commonly used of 60% of the body weight of subjects (assuming 60% of weight being total body water into which these metabolites distribute)^19–21^. As would be expected, total body water PA/TYR metabolites showed no difference between baseline and 4 weeks of study in those who received no nitisinone. In those patients that received nitisinone there was a significant increase in total body water ALL metabolites post-nitisinone at all doses. In those that received 4 and 8 mg nitisinone there was a higher increase in total body water ALL metabolites post-nitisinone, compared with 1 and 2 mg of nitisinone, suggesting a more complete block of flux through the pathway. In addition there was little difference between 4 and 8 mg in terms of total body water metabolites suggesting that HGA formation was maximally inhibited.

### Making sense of aggregated metabolites

The sum of 24-hour urine and total body water PA/TYR metabolites (_ALL_PA + _ALL_TYR + _ALL_HPPA + _ALL_HPLA + _ALL_HGA), *i*.*e*. all excreted and retained metabolites, demonstrated a significant increase post-nitisinone. Again, in those that received 4 and 8 mg nitisinone there was a higher increase in ALL PA/TYR metabolites post-nitisinone, compared with 1 and 2 mg of nitisinone.

**Figure 3:**
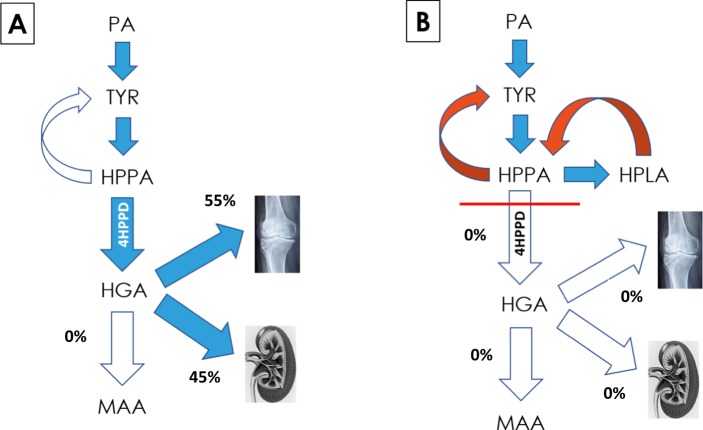
Schematic of the effects of nitisinone on HGA. HGA accumulates in AKU due to a deficiency of homogentisate dioxygenase, with spill over into urine and also accumulation in the body as pigment. In AKU (Panel A), circulating HGA is in low concentration relative to urinary excretion and therefore the urinary excretion is dominant. However, data post-nitisinone shows that approximately 55% of TYR flows towards pigment formation and only 45% is excreted in the urine, following 8 mg nitisinone daily for 4 weeks. Panel B shows the complete inhibition of HGA excretion and HGA deposition and the suggested mechanism for metabolite generation post high dose nitisinone treatment. (4HPPD: 4-hydroxyphenylpyruvate dioxygenase; PA: phenylalanine; TYR: tyrosine; HPPA: 4-hydroxyphenylpyruvate; HPLA: 4-hydroxyphenyllactate; HGA: homogentisic acid; MAA: maleylacetoacetic acid). Kidney image is provided free of copyright under Creative Commons CC0 1.0 licence from Pixabay; permission is provided without attribution.

Surplus PA and TYR is degraded to fumarate and acetoacetate in healthy individuals, principally in the liver, and to a smaller extent in the kidneys, the only two organs where significant expression of HGD is observed. The quantity of TYR that is degraded to fumarate and acetoacetate is difficult to quantify. The metabolic block in AKU results in interruption of the pathway with accumulation of HGA, and it has been assumed that this allows a better indication of TYR flux, reflecting dietary intakes. However, this assumption should be tempered by the fact that HGA also binds to tissue and forms ochronotic pigment, not measured in the usual 24 hour urine collection and quantification. Nitisinone blocks the formation of HGA thereby decreasing HGA concentration but leading to increases in proximal metabolites. Since the decrease in daily urine HGA was balanced by a proportionate increase in urine HPPA and HPLA, the cause of the marked increase in total body water metabolites post-nitisinone is surprising and unexpected. The most plausible explanation for the marked increase in TYR metabolite flux post-nitisinone is that these metabolites were previously being directed down the HGA – benzoquinone pathway to form the ochronotic pigment (Fig. 3).

There was a 5, 81, 85, 126 and 132% increase in metabolites compared with pre-nitisinone or baseline values, at doses of 0, 1, 2, 4 and 8 mg of nitisinone respectively, following 4 weeks of nitisinone therapy.

The discrepancy in terms of the elevated quantity of ‘new’ metabolites post-nitisinone is greater at higher doses of nitisinone and suggests that higher doses (4 and 8 mg) compared with the lower doses (1 and 2 mg), are more effective in blocking the metabolite pathways, including those directed at ochronotic pigment formation. These results are also consistent with a more complete inhibition of ochronosis by 4 and 8 mg doses, suggesting more appropriate doses of nitisinone to be employed in practice.

### Ochronosis (conversion of HGA to pigment) -? The evidence

There is a paucity of literature on how ochronosis occurs in AKU and any pathways associated with the pigment formation. Early literature proposes a “pathway” whereby HGA is oxidised to an ochronotic pigment, via the intermediary benzoquinone acetic acid (BQA)^5^; all the classic characteristics of a pathway are described whereby molecules are converted via enzymes, with various cofactors present. Chemistry of HGA conversion to BQA has been described^22^ and Raman spectroscopy^23^, LC-TOF/MS and NMR spectroscopy and spectrophotometric techniques have confirmed the HGA conversion and presence of BQA^24^. To date however, the polyphenol oxidase enzyme suggested by Zannoni *et al*.^5^, to be involved with the conversion to ochronotic pigment has not been isolated or localised in AKU.

Wolff *et al*.^25^ treated two adults and three infants with AKU with high doses of ascorbic acid and studied the effect on excretion of HGA and BQA. The purpose was to determine whether the concentration of BQA was decreased in bodily fluids. Disappearance of BQA was shown in adults whereas the level of HGA in urine did not change. They concluded that this could have relevance to the pathogenesis of ochronotic arthropathy and is evidence that metabolite flux towards ochronosis is important in AKU. However, the quantity of HGA converting to BQA was not described and no systematic measurements of PA, TYR, HPPA and HPLA were carried out in urine or in serum. Lustberg *et al*.^26^ presented evidence that ascorbic acid at high doses decreases binding of ^14^C HGA in connective tissues of experimental rats with AKU. On this basis, daily therapy with ascorbic acid has been used in practice for a long time without much clinical evidence to support its efficacy. More recent evidence from an *in vitro* model have demonstrated that reducing agents such as ascorbic acid and other antioxidants can prevent HGA oxidation to BQA and subsequent ochronotic pigment formation^27,28^. Factors involved in pigment deposition have been debated and eloquently reviewed by Millucci *et al*.^29^, where pigment deposition was linked to collagen and amyloid fibrils, but were not essential in all cases reviewed, as pigment was also found intracellularly in cells of the submandibular gland. In addition, BQA proteins induced by the presence of HGA *in vivo* have been identified^30^. Key to all mechanisms was accumulation and or local production of oxidised HGA, also known as BQA.

### Proportion of metabolites progressing to pigment formation

The ratio of pre-nitisinone ALL metabolites to post-nitisinone ALL metabolites reflects the proportion of metabolites pre-nitisinone compared with total (*i*.*e*. post-nitisinone more metabolites became apparent). If pHPPD is not maximally inhibited, there could still be flux down the HGA pathway. The data indicates that at 4 and 8 mg of nitisinone there is near-maximal inhibition with similar quantities of metabolites at these two doses.

When expressed as a percentage of total baseline value of zero dose nitisinone, 97% of the sum of metabolites were present at baseline; this lack of change supports the robustness of the quantitative approach presented herein. At doses of 1 and 2 mg of nitisinone, a mean of 58 and 57% of total metabolites were visible at baseline, respectively, suggesting that two fifths of total metabolites were passing through, potentially towards ochronotic pigment formation. At doses of 4 and 8 mg, 48 and 45% of total metabolites were visible at baseline, respectively, suggesting that approximately 50% of metabolites were undetected and potentially being shunted towards ochronotic pigment formation (Fig. 3). It is worth emphasising that in addition to blocking HGA-pigment or adsorption of HGA to connective tissue, partial reversal of pigment is a possibility. Further long-term studies are needed to explore the possibility of reversal of pigmentation. Patients using nitisinone spontaneously report lightening of pigmentation in ears and eyes, but objective confirmation is needed if reversal of pigment is to be definitively proven. If reversal is shown to occur, it might further explain why there is such marked increase in TYR pathway metabolites post-nitisinone.

The measure of ALL PA/TYR pathway metabolites is a reflection of the daily TYR flux since PA, TYR, HPPA, HPLA and HGA are equimolar. The quantities of TYR equivalent post-nitisinone in the form of ALL metabolites was estimated to be 7.2, 14.7, 13.3, 17.7 and 15.3 grams at 0, 1, 2, 4 and 8 mg doses. This is the first time it has been possible to quantify the TYR pathway in this manner in AKU.

In conclusion, the level of tyrosine metabolite flux within this pathway was unexpected. The adjustment of circulating metabolites to derive total body water metabolites has revealed a massive increase in the quantity of metabolites with a purported large flux towards pigment formation. There is an enormous burden of ochronotic pigment in ageing patients with AKU^31^, and the magnitude of the tyrosine metabolites within this pathway are in keeping with this even though the amount of ochronotic pigment in a human body has yet to be quantitated.
